# Prevalence of Intestinal Parasitic Infections and Associated Factors Among Food Handlers in Wolaita Sodo University Students Caterings, Wolaita Sodo, Southern Ethiopia: A Cross-Sectional Study

**DOI:** 10.3389/fpubh.2019.00140

**Published:** 2019-06-04

**Authors:** Wondimagegn P. Kumma, Wubshet Meskele, Amha Admasie

**Affiliations:** School of Public Health, Wolaita Sodo University, Sodo, Ethiopia

**Keywords:** intestinal parasitic infections, food handlers, associated factors, Wolaita Sodo University, Southern Ethiopia

## Abstract

**Background:** Intestinal Parasitic Infections (IPIs) are among the most prevalent diseases in the world, particularly in developing countries with low socio-economic and poor living conditions. From the estimated one third global population infected by intestinal parasitic infections; the majority lives in tropical and sub-tropical parts of the world. These diseases are highly prevalent in Ethiopia. However, its magnitude in the context of Wolaita Sodo University, where a large number of students reside in a campus is not studied. Therefore, the objective of this study was to assess the prevalence and factors associated with intestinal parasitic infections among food handlers in students' caterings of Wolaita Sodo University, Southern Ethiopia.

**Methods:** Institutional based cross sectional study was conducted among food handlers working in Wolaita Sodo University students' caterings' from January 10 to February 10, 2016. The study was undertaken among 233 food handlers by using simple random sampling technique. Data were collected using structured and pretested questionnaire; observation of food handlers while working and microbiological laboratory investigations. Bivariate and multivariate analyses were used to assess the association between covariate and the outcome variables. Adjusted Odds Ratio (AOR) with 95% confidence interval (CI) was considered to ascertain the significance of the association.

**Results:** The mean age of the study participants was 27.2 (±6.4 SD). The overall prevalence of intestinal parasitic infections was 23.6%, with 95% CI of 18.2% to 29.1% of which 12.4% was due to amoeba cyst. Untrimmed fingernail with AOR (95% CI) 2.06 (1.06, 4.00) and regular hand washing habit without soap before food handling with AOR (95% CI) 2.68 (1.25, 5.73) were found to be significantly associated with intestinal parasitic infections.

**Conclusions:** The prevalence of intestinal parasitic infections was high among Wolaita Sodo University food handlers. Untrimmed fingernail and hand washing habit without soap before food handling were found to increase odds of intestinal parasitic infections. The university may need to consider interventions recommended accordingly.

## Background

Intestinal Parasitic Infections (IPIs) are among the most prevalent diseases in the world, particularly in developing countries with low socio-economic and poor living conditions. (1–4). From the estimated one third global population infected by intestinal parasitic infections; the majority lives in tropical and sub-tropical parts of the world (5, 6). These infections are endemic in some countries of Africa, Southeast Asia, South America and Eastern Europe (2, 4, 7).

The epidemiology of intestinal parasitic infections in the world varies from parasites to parasites. *Entamoeba histolytica, Ascaris lumbricoides (Roundworm), Ancylostoma duodenale (hookworm), and Trichuris trichiura (Whipworm)* are the most common. According to the World Health Organization (WHO) estimates, globally there are 800–1,000 million *A. lumbricoides*, 700–900 million Hookworm, 500 million *T. trichiura*, 200 million *Giardia intestinalis*, and 500 million *E. histolytica/dispar* cases (8–10). Despite the efforts made to control IPIs, the diseases are continuing to be the leading causes of mortality and morbidity in the world (2, 4, 11, 12).

Ethiopia is one of the countries in Africa with high prevalence of intestinal parasitic infections. Based on the studies conducted in Ethiopia; one third of the population is estimated to be infected with *Ascaris lumbricoides*, one quarter is infected with *Trichuris trichiura* and one in eight lives with hookworm (4, 11, 13–17). Thus, Ethiopia has the second highest ascariasis, the third highest hookworm, and the fourth highest trichiuriasis infections in Sub-Saharan Africa (4, 11, 13–17).

The health status and hygienic practices of the food handlers are the major determinants of food contamination in developing countries where there is a poor regulatory system for food hygiene. Because of urbanization and expansion of large institutions; eating and drinking from food caterings are becoming common practices in developing countries like Ethiopia (4–6, 11). Often, food handlers in developing countries, including Ethiopia are employed without proper screening for hygiene related infectious diseases (4, 11, 18). The rapid growth of public universities in Ethiopia in the past two decades, urging to ensure hygienic food handling and preparation practices to safeguard the health of students getting food and drink services in a mass. Meals served in caterings' of higher learning institutions can cause food borne disease outbreaks. A few local studies conducted among food handler's revealed high prevalence of IPIs and unsatisfactory practices of food hygiene measurements (19). However, most studies were old and no similar studies had been conducted in the study area. Therefore, this study aimed at assessing prevalence and factors associated with IPIs among food handlers in the context of Wolaita Sodo University (WSU).

## Methods

### Study Setting and Period

Institutional based cross sectional study was conducted at WSU, Wolaita Sodo, Southern Ethiopia from January 10 to February 10, 2016. WSU enrolled a total of 11,235 regular students in the 2016 academic year in its two campuses located at Sodo town. The university supplies, food and drink services for its students residing in two campuses located in Wolaita Sodo town. There are 558 food handlers working in the students' caterings, of which 144 were males and 414 were females. WSU has only two campuses (Gandaba and Ottona) only.

### Source Population

The source population was all food handlers who were working in the students' Caterings of WSU located in Wolaita Sodo town.

### Study Population

The study population was randomly selected food handlers working in the students' caterings of WSU. Food handlers engaged in food preparation, serving and cleaning regardless of their employment status were included in the study. Whereas, those who were on annual leave during the data collection time and those who were taking anti-parasitic drugs in the past two to three weeks prior to the data collection were excluded from the study.

### Sample Size and Sampling Technique

The sample size was calculated based on a study in Mekelle university showed that IPIs has been reported in 49.4% of food handlers. Assuming, 95% Confidence Interval (CI), 5% margin of error and 10% non-response rate using single population proportion formula, finite population correction factor for a population <10,000 was used to calculate final sample size (19). Hence, a total of 251 samples were determined. Study participants were selected using simple random sampling technique after entering alphabetic ordered lists of food handlers into Statistical Software for Social Sciences (SPSS) version 20. The samples were distributed between two campuses proportional to their size.

### Data Collection Instruments and Procedures

A structured and pretested questionnaire was used to collect data related to socio-demographic, economic, personal and environmental characteristics. Variables such as age, sex, monthly income, personal hygiene, regular medical checkup, waste management and accessibility to safe water supply were included in the questionnaire. The questionnaire was prepared in English and translated into local language. Data were collected by two Environmental Health Officers who speak the local language. Trained staffs collected the data. Training for data collectors and supervisor was given for 3 days. Supervision was made by Environmental Health Officer who has experience of research work and familiar with the study area. Pretest before the actual data collection was carried out on food handlers working at Wolaita Sodo Agricultural Vocation Training College with a size equivalent to 5% of the study subjects.

The stool collection and microscopic investigation were conducted by three Medical laboratory technologists. Triplicate smears from the collected sample of every study subjects were prepared. To ensure consistency of measurements every smear examination was carried out and confirmed by two laboratory technologists. Stool specimens were collected from food handlers with labeled wide-mouthed clean plastic cups using clean wooden applicator stick at WSU Teaching and Referral Hospital Medical Laboratory (20, 21). From each study subjects about 2 gm of stool was collected. The stool samples were processed in WSU Teaching and Referral Hospital Medical Laboratory using WHO standard and calibrated instruments with a direct microscopic technique to detect cysts, trophozoites, eggs, and larva of intestinal parasites (20). Stool examinations were performed using the formol-ether concentration technique. Both the 10× and 40× objectives were used to detect eggs and larvae of helminthes and cysts and trophozoites of protozoan parasites. Iodine solution was used to detect and identify cysts of protozoan parasites (20, 21). The results of the stool examination and observation of the working environment were recorded using checklists.

### Data Analysis

Data were checked for completeness and consistency. Coded data were entered using EpiData version 3.1 software and exported to SPSS version 20 for Windows (SPSS, Chicago, IL, USA) for cleaning and analysis. Descriptive summaries were calculated to determine the prevalence of intestinal parasitic infections. Positive result of IPIs coded = 1 and negative results coded = 0 in a logistic regression analysis. Based on the existing evidence, the least contributing factor to the dependent variable was used as reference category. Variables with *P*-value <0.25 in bivariate analysis were included in the multivariate analysis. Adjusted Odds Ratio (AOR) with 95% CI was considered to ascertain the significance of the association at *p*-value < 0.05.

## Results

### Socio-Demographic Characteristics

A total of 233 food handlers were participated with a response rate of 92.8%. Most of the food handlers are female dominant in WSU, hence the male to female ratio of the respondents was 0.25. The mean age of the study participants was 27.2 (±6.4 SD). About 68 (29.2%) participants were educated at the level of college and above. The mean monthly income of respondents was 825 (± 346 SD) Ethiopian Birr (ETB) ranged from 500 to 3,000 ETB (Note: 1USD = 29.00 ETB in May, 2019) (Table 1).

**Table 1:** Socio-demographic characteristics of the food handlers worked in the students' caterings of WSU, Wolaita Sodo, South Ethiopia from January 10 to February 10, 2016.

**Characteristics (*n* = 233)**	**Categories**	**Frequency**	**Percent (%)**
Campus	Gandaba Ottona	215 18	92.2 7.8
Sex	Male Female	46 187	19.7 80.3
Age in years	15–29 30–44 45+	159 73 1	68.3 31.3 0.4
Job position	Cook Cleaner/ waiter Others^*^	143 75 15	61.4 32.2 6.4
Educational status	Primary school Secondary school College and above	53 112 68	22.7 48.1 29.2
Average monthly income	≤ 1,000 ETB 1,001–1,500 ETB 1,501+ ETB	205 22 6	88.0 9.4 2.6
Work experience	< 1 year 1–2 years ≥2 years	21 36 176	9.0 15.5 75.5

* *Others: Shift leaders and wood cutters*.

### Personal Hygiene Practices of Food Handlers

From the total food handlers 113 (48.5%) observed using an outer protective coat or gown during observational assessment of food handlers at work. During observation of finger nail status, 103 (44.2%) food handlers found to have their fingernails untrimmed. About 158 (67.8%) food handlers washed their hands after toilet with soap, while the rest washed only with water. The magnitude of hand washing with soap habit before food handling was 189 (81.1%) (Table 2).

**Table 2:** Personal hygiene practices of food handlers worked in the students' caterings of WSU, Wolaita Sodo, South Ethiopia from January 10 to February 10, 2016.

**Characteristics (*n* = 233)**	**Categories**	**Frequency**	**Percent (%)**
Outer protective coat/gown	Observed Not observed	113 120	48.5 51.5
Hair cover	Observed Not observed	109 124	46.8 53.2
Cleanness of outer garments	Kept Not kept	165 68	70.8 29.2
Finger nail status	Trimmed Not trimmed	130 103	55.8 44.2
Presence of ring	Observed Not observed	43 190	18.5 81.5
Nail painted	Yes No	195 38	83.7 16.3
Hand washing habit with soap after toilet	Yes No	158 75	67.8 32.2
Hand washing habit after touching dirty materials with Soap	Yes No	143 90	61.4 38.6
Hand washing habit with soap before food handling	Yes No	189 44	81.1 18.9
Regular medical checkup	Checked Not checked	132 101	56.7 43.3
Hygiene education	Trained Not trained	21 212	9.0 91.0

### Knowledge of Food Handlers

Out of the total participants interviewed for knowledge assessment, 225 (96.6%) knew about person to person transmission of IPIs. On the other hand 232(99.6%) knew washing hands before handling food reduce the transmission of IPIs (Table 3).

**Table 3:** Knowledge of food handlers worked in the students' caterings of WSU, Wolaita Sodo, South Ethiopia from January 10 to February 10, 2016.

**Knowledge**	**Categories**	**Frequency**	**Percent (%)**
IPI is transmittable from person to person	Yes I don't know	225 4	96.6 3.4
Cleaning of utensils decrease the transmission of IPIs	Yes I don't know	231 2	99.1 0.9
Washing hands before handling food reduce the transmission of IPIs	Yes I don't know	232 1	99.6 0.4
Proper clothing/hair cover, gown/ reduce the transmission of IPIs	Yes	233	100.0
Utilization of latrine decreases the transmission of IPIs	Yes	233	100.0
Use of potable water decreases the transmission of IPIs	Yes	233	100.0

### Prevalence of Intestinal Parasitic Infections

The overall prevalence of IPIs among the food handlers in the students' catering center of WSU was 23.6% (95% CI: 18.2–29.1%). Among all participants, 55(23.6%) were positive for at least one intestinal parasite. Amoeba cyst was the predominant parasite 29(12.4%) followed by Amoeba trophozoites 10 (4.3%) (Table 4).

**Table 4:** Prevalence of IPIs identified from food handlers worked in the students' caterings of WSU, Wolaita Sodo, South Ethiopia from January 10 to February 10, 2016.

**Parasite species Isolated (*n* = 233)**	**Gandaba campus (No.)**	**Total (%)**
Protozoan parasites	47	20.2
E—histolytica	39	16.7
Amoeba cyst	29	12.4
Amoeba trophozoites	10	4.3
G—lamblia	12	5.2
Giardia cyst	5	2.2
Giardia trophozoites	7	3.0
Helminthiasis	6	2.6
Tape worms	3	1.3
Hookworms	3	1.3
Double parasites^*^	2	0.8
Over all parasite rate /IPIs/	55	23.6

* *Amoeba and Giardia*.

### Factors Associated With Intestinal Parasitic Infections

Socio-demographic and hygiene related factors such as work experience and finger nail status were entered into binary logistic regression and then variables showing *p* value < 0.25 were re-entered into multivariate logistic regression to control for confounding. From the binary logistic regression analysis, hand washing habit with soap before food handling with Crude Odds Ratio (COR) (95% CI) of 2.84 (1.41–5.72) was positively associated with intestinal parasitic infections. However, in the binary logistic regression variables such as work experience, finger nail status, educational status, hand washing habit with soap after touching dirty materials, nail painted and cleanness of outer garments were not associated with intestinal parasitic infections (Table 5).

**Table 5:** Factors associated with IPIs among food handlers worked in students' caterings of WSU, Wolaita Sodo, South Ethiopia from January 10 to February 10, 2016.

**Variables**	**Categories**	**Positive**	**Negative**	**COR (95%CI)**	**AOR (95%CI)**
Work Experience	< 1 year	1	20	0.16(0.02–1.19)	0.13(0.016–1.05)
	1–2 years	11	25	1.36(0.62–2.99)	1.34(0.58–3.12)
	≥2 years	43	133	1	1
Hand washing habit with soap before food handling	No	18	26	2.84(1.41–5.72)	2.67(1.11–6.42)^*^
	Yes	37	152	1	1
Finger nail status	Not trimmed	30	73	1.73(0.94–3.17)	1.93(1.01–3.67)^*^
	Trimmed	25	105	1	1
Educational Status	Primary	14	39	1.68(0.70–4.01)	1.64(0.63–4.26)
	Secondary	29	83	1.63(0.77–3.46)	2.02(0.91–4.54)
	College	12	56	1	1
Hand washing habit with soap after touching dirty materials	Yes	30	113	1	1
	No	25	65	1.45(0.78–2.67)	0.78(0.36–1.71)
Nail painted	Observed	13	25	1	1
	Not observed	42	153	0.53(0.25–1.12)	0.46(0.21–1.06)
Cleanness of outer garments	kept	35	130	1	1
	Not kept	20	48	0.65(0.34–1.23)	1.40(0.69–2.86)

* *Significance factor at P < 0.05*.

On multivariate analysis hand washing habit with soap before food handling and finger nail status showed a significant association with intestinal parasitic infections. There is a 93 % increased odds of developing intestinal parasitic infections among food handlers who had untrimmed finger nail with AOR (95% CI) of 1.93 (1.01, 3.67). Food handlers who had no habit of washing their hands with soap before food handling were 2.67 times more likely to be infected with intestinal parasitic infections than their counterparts with AOR (95% CI) of 2.67 (1.11, 6.42) (Table 5).

## Discussion

The spread of intestinal parasitic infections (IPIs) through food handlers is a common and persistent problem worldwide (4, 11, 13–17). Intestinal parasitic infections are among the major health problems in developing countries like Ethiopia (22). The spread of intestinal parasitic infections through food handlers can cause epidemics in the public and students in the university. Therefore, this study was undertaken to assess the prevalence and associated factors of intestinal parasitic infections among food handlers in students' caterings of Wolaita Sodo University. The prevalence of intestinal parasitic infections in this study was found to be 23.6%.

The prevalence of IPIs observed in this study is comparable with the findings from Eldoret Municipality, Kenya (23.7%) (23) and Gonder in Ethiopia (29.1%) (24). However, it is higher than the findings reported from Western Iran (9%) (25), North India (7%), Sari (26), Northern Iran (15.5%) (27). The observed discrepancy might be due to differences in climatic conditions, geographic locations and socio-economic features of the populations. The finding reported in this study is lower than the findings obtained from other studies conducted in Ethiopia; Addis Ababa (45.3%) (20), Hawassa (63%) (28), Mekelle (49.4%) (19), Jimma (58.4%) (22), Yebu (44.1%) (6), Bahir Dar (41.1%) (29), and Arba Minich (36%) (16). The difference might be due to socio-demographic features and geographic locations of the populations.

The rate of double infections was 0.8%. This is relatively similar to the findings from Arba Minich University students' caterings in Southern Ethiopia (0.6%) (16) and Gondar University students' caterings(1.6%) in North-western Ethiopia (24). While it is lower than the finding from Mekelle university students' caterings(5.2%) in Northern Ethiopia (19). Availability and accessibility of medical checkups at Wolaita Sodo University and socio-demographic features might be some of the reasons for the difference.

In this study, the predominant parasitic infection was *Amoeba cyst* with a prevalence of 12.4%. This is consistent with the finding from Bahir Dar, North-western Ethiopia, 10.2% (29, 30). The prevalence of hookworm was 1.3%, which is in consistent with the findings from Jimma, South-western Ethiopia (2.9%) (31), and Addis Ababa University (2.1%) (20).

Untrimmed fingernail and hand washing habit without soap before food handling were found associated with IPIs. This is similar with the findings from Arbaminch University; where, untrimmed finger nail, hand washing practice after toilet and hand washing before food handling were identified as predictors for IPIs (16). This evidence also supported with the study conducted from Mekele University; where, hand washing habit with soap before food handling and utilization of soap were found determinants for IPIs (19). Both of the study, participants were from university cafeteria food handlers. In either of evidences, untrimmed fingernail can serve as a vehicle for the transmission of intestinal parasitic infections and washing hands with soap before handling food could remove parasites. The study from Addis Ababa University has found no association between untrimmed finger nail and hand washing habit with soap before food handling; and intestinal parasitic infections (20). Examination of food handlers' fingernail contents for ova or parasites is one way of identifying possible contamination of food; however, the current study did not include the assessment of parasite in the fingernail contents. The data of this study are limited to Wolaita Sodo University; therefore, we recommend further study that include other universities in the country. Since the study, design is cross-sectional; it cannot establish temporal relationship between variables; therefore, we recommend intervention study in the same population.

## Conclusions

The present study demonstrated a high prevalence of IPIs among food handlers working in Wolaita Sodo University. The study revealed that fingernail determines the prevalence of IPIs, similarly handwashing with soap before food handling was additional determinants of IPI in food handlers. It identified fingernail status and hand washing habit with soap before food handling as determinants of IPIs among food handlers in Wolaita Sodo University. Therefore, enforcement of standard hygienic and sanitary practices by the university was recommended to improve personal hygiene of the food handlers and sanitary facilities of the caterings.

## Ethics Statement

The study was conducted after obtaining ethical clearance from WSU College of Health Sciences and Medicine institutional ethical review committee. Letter of cooperation obtained from the university was submitted to the students' caterings administration. Informed oral consent was obtained from the participants of the study. Participants found positive for intestinal parasitic infections were treated at WSU Teaching and Referral Hospital. Information obtained from the study participants was kept confidential. Information's were kept locked in office. Any personal information never pass to the third party.

## Author Contributions

WK, WM, and AA designed the study, responsible for supervision, and wrote the manuscript. WM collected data and analysis.

### Conflict of Interest Statement

The authors declare that the research was conducted in the absence of any commercial or financial relationships that could be construed as a potential conflict of interest.

## Abbreviations

AOR: Adjusted Odds Ratio
CI: Confidence Interval
COR: Crude Odds Ratio
ETB: Ethiopian Birr
IPIs: Intestinal Parasitic Infections
SPSS: Statistical Package for Social Sciences
WHO: World Health Organization
WSU: Wolaita Sodo University.
